# Evidence for sequence biases associated with patterns of histone methylation

**DOI:** 10.1186/1471-2164-13-367

**Published:** 2012-08-02

**Authors:** Zhong Wang, Huntington F Willard

**Affiliations:** 1 Genome Biology Group, Duke Institute for Genome Sciences & Policy, Duke University, 101 Science Dr. CIEMAS 2376, Durham, NC, 27708, USA; 2 DOE Joint Genome Institute, Walnut Creek, CA, 94598, USA

## Abstract

**Background:**

Combinations of histone variants and modifications, conceptually representing a histone code, have been proposed to play a significant role in gene regulation and developmental processes in complex organisms. While various mechanisms have been implicated in establishing and maintaining epigenetic patterns at specific locations in the genome, they are generally believed to be independent of primary DNA sequence on a more global scale.

**Results:**

To address this systematically in the case of the human genome, we have analyzed primary DNA sequences underlying patterns of 19 different methylated histones in human primary T-cells and patterns of three methylated histones across additional human cell lines. We report strong sequence biases associated with most of these histone marks genome-wide in each cell type. Furthermore, the sequence characteristics for such association are distinct for different groups of histone marks.

**Conclusions:**

These findings provide evidence of an influence of genomic sequence on patterns of histone modification associated with gene expression and chromatin programming, and they suggest that the mechanisms responsible for global histone modifications may interpret genomic sequence in various ways.

## Background

The basic unit of eukaryotic chromosomes is the nucleosome, comprised of DNA wrapped around a histone octamer complex [1]. Nucleosomes can adopt distinct chromatin structures, associated with specific post-translational modifications of histone proteins at their N-terminal tails [2]. Such histone modifications can be stably maintained through cell divisions and are strong candidates to serve as marks for epigenetic regulation. Epigenetic modifications either influence the accessibility of *cis*-regulatory elements in genomic DNA or recruit chromatin-binding proteins to regulate gene expression.

The histone code or epigenetic code theory proposes that the combinatorial nature of histone modifications and histone variants represents information that greatly extends the content and display of genetic information alone [3-8]. As one approach to testing this model, a number of studies have begun to define genome-scale maps of various histone modifications and other chromatin constituents and to relate such maps to cellular phenotypes in organisms from yeast to human [9-11]. Significantly, the ENCODE project reported a high-resolution epigenetic landscape of 1% of the human genome that could be used to accurately predict gene expression in a variety of cell types [12] and increasing amounts of ENCODE whole-genome data are becoming available on genome browsers [13].

**Figure 1:**
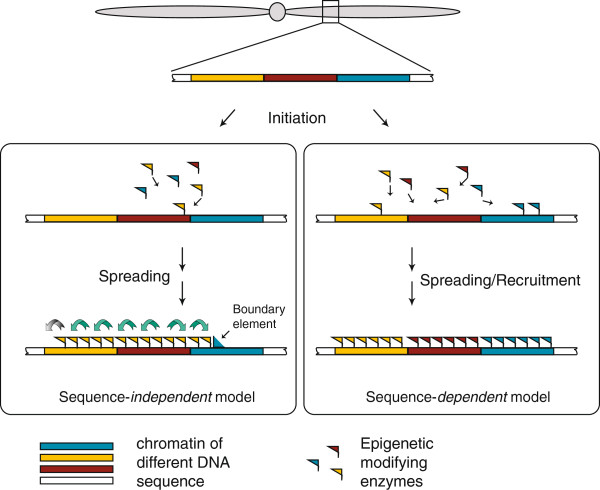
**Alternative models for the formation of epigenetic domains in complex genomes.** In a sequence-independent model (*left*), histone-modifying enzymes respond to local signals, but subsequent establishment and maintenance of epigenetic modifications within larger domains are largely independent of the overall sequence composition of that domain. The extent of the domain, however, might be determined by other local sequence-specific features such as boundary elements. In a sequence-dependent model (*right*), recruitment and spreading of histone-modifying enzymes is guided by features of the primary genomic sequence throughout the domain, with different sequence features specifying or influencing different modifications across domains genome-wide.

While much has been done to explore the nature of the histone code and how it is “read”, little is known about how and where the code is written in the first place [14]. A prevailing hypothesis is that histone- and DNA-modifying enzymes, although lacking DNA sequence specificity themselves, can be targeted to specific sites by *trans*-acting co-factors such as transcription factors that bear sequence specificity or even by various classes of RNA, including noncoding RNAs and small RNAs [15] (Figure 1). Histone or DNA modifications at these sites would then spread in *cis* until they encounter barrier or boundary elements defined by patterns of histone replacement or by CTCF binding to form coherently marked epigenetic domains [16-18]. The fact that epigenetic modifications can encompass large regions of genomic DNA and that epigenetic marks display certain plasticity further supports this hypothesis [19]. Under this model, the body of epigenetic domains should be largely independent of primary DNA sequence (Figure 1, *left*).

Alternatively, however, it is possible that the genomic sequence could influence different epigenetic domains and that primary DNA sequence itself could thus bias their formation and/or maintenance on a genome-wide basis (Figure 1, *right*). This hypothesis can account for chromatin state persistence across cell cycles and even through generations [20]. Consistent with the predictions of this model, several recent studies have demonstrated that some epigenetic marks, such as the position of methylated CpG islands [21-23] or the presence of trimethylated forms of histone H3 at lysine 4 (H3K4me3) or at lysine 27 (H3K27me3) [24], are correlated with particular features of complex genomes. Furthermore, binding of CTCF to *cis* elements has been shown to form well-positioned nucleosome arrays around them, providing a potential mechanistic link between primary genome sequence and chromatin state [25]. Recently, it has been shown that mammalian chromosomes are organized into megabase-size domains stable across cell types and conserved across species, with specific genomic features marking their boundaries [26]. While these findings support the genomic influence model in specific local instances, it remains a question whether the histone code, consisting of many different types of histone modifications and variants, is associated with primary DNA sequence genome-wide.

In this study, we have tested this hypothesis for the human genome by investigating correlations of genomic regions associated with a wide range of methylated histones with the underlying DNA sequence. To achieve this, we used high-resolution, genome-wide epigenetic maps and applied a machine learning approach called Support Vector Machine (SVM, reviewed in [27]), which can be used successfully to computationally predict other epigenetic states [22,28]. Like other machine learning algorithms, SVM has the ability to recognize patterns in a given dataset (used for training), and the resulting models can then be tested with previously unseen examples and new predictions can be made accordingly. Thus, an association between genome sequence and epigenetics can be tested by investigating whether or not primary sequence alone is sufficient to predict the genomic location of the histone code.

## Results

### Genomic sequence alone discriminates regions enriched or depleted for most methylated histones in human CD4+ cells

To investigate whether histone marks in general are associated with underlying genomic sequence, we analyzed a dataset containing the profiles of 19 different methylated histones from genome-wide ChIP followed by deep sequencing experiments (ChIP-Seq) in human CD4+ T-cells [29]. (Of the 20 marks reported by Barski *et al.*[29], H3K29me2 was excluded because of low sequence coverage in the original dataset.) We asked whether genomic sequence could distinguish between regions that are enriched or depleted for these histone marks in this cell type.

**Figure 2:**
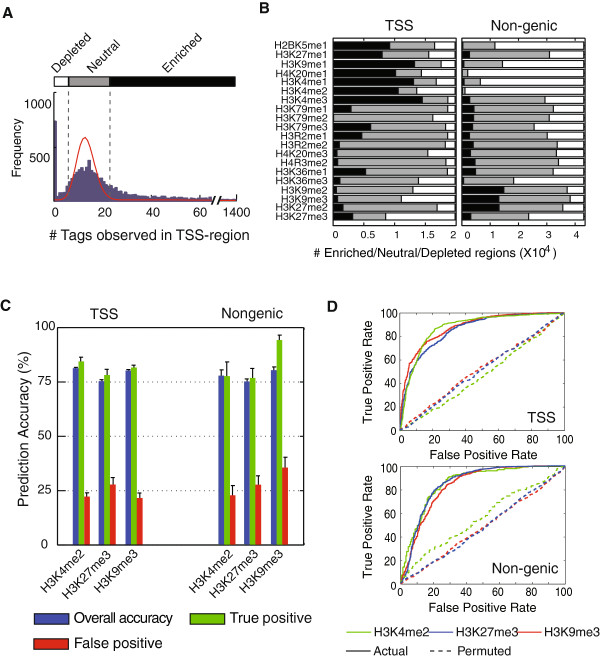
**Classification of epigenetic marks in human CD4+ cells. (A)** The frequency of sequence tags in a typical ChIP-SEQ experiment (H3K4me3) in 4 kb regions [36] and a simulated null distribution (red line). Vertical lines represent a false discovery rate (FDR) of 1% from each side in the null distribution (Depleted and Enriched), leaving the rest as (Neutral). (**B)** Based on the criteria in (**A)**, the number of selected enriched (dark gray), depleted (white), or neutral (medium gray) regions for each histone mark. (**C)** The SVM classification of three representative methylated histone marks. Error bars represent standard deviations (s.d.) of 10 SVM predictions. (**D)** Receiver Operating Characteristic (ROC) curves for the classification of three representative methylated histone marks (solid lines) and the corresponding curves for classification with randomized sample labels (dotted lines).

To select such regions for analysis, we first simulated a null distribution of the tag frequency (tags/region) for each mark, from which we determined selection criteria that limit the false discovery rate to only 1% at each extreme (one example is shown in Figure 2A). Based on these criteria, we identified ±2 kb regions that surround either transcription start sites (TSS) or non-genic, non-repetitive regions in the genome as significantly “enriched” or “depleted” relative to the null distribution (or “neutral” if the frequency of the histone mark was not different from the null distribution) for each of 19 methylated histone states. Overall, TSS and non-genetic regions display very different epigenetic profiles (Figure 2B and Additional file 1: Table S1). Many TSS regions are significantly enriched for H3K4me1-3, H3K9me1, H3K27me1, H4K20me1 and H2BK5me1, while many non-genic regions are notably enriched for H3K27me2 and H3K9me2-3 but depleted for H3K4me2 and H4K20me1 (Figure 2B). Given these differences, we analyzed TSS and non-genic regions separately throughout to avoid potential inherent differences between these two types of sequences. Sequence information for each region was captured by the content of all occurring *k*-mers (*k* = 1,2,3,4,5).

To avoid potential over-fitting problems associated with many machine learning approaches when the number of variables greatly exceeds the number of samples [30], we used a large sample size (up to 1,000 depleted and 1,000 enriched regions randomly selected from the genome, Additional file 2: Table S2) for each methylated histone and performed SVM classification experiments. Each classification experiment involved training a SVM model using 1,000 regions randomly sampled from the above 2,000 regions, and testing its prediction performance on the rest. This classification procedure was reiterated ten times to minimize sampling bias. If genome sequence has no influence on histone modification placement, SVM models should randomly assign sequences to be “enriched” or “depleted” (thus, prediction accuracy =50%). However, for all 19 histone marks in TSS regions and for 14 histone marks in non-genic regions, the prediction accuracy of the SVM models was significantly greater than 50% (p < 0.01), with over 75% accuracy in most cases (Additional file 3: Table S3). Three specific examples, the classifications for a euchromatin mark H3K4me2 and two heterochromatin marks, H3K9me3 and H3K27me3, are shown in Figure 2C. Classification performance of these examples was further visualized using their Receiver Operating Characteristic (ROC) curves (Figure 2D). Specifically, the Area Under Curve (AUC) values from these ROC curves are around 0.85, suggesting the models have very high discrimination power (AUC = 1.00 means perfect classification while 0.50 means none). In contrast, AUC values drop to ~0.50 and classification ability is completely lost when models were trained without the correct enrichment/depletion information among the training samples (“permuted” in Figure 2D). Thus, these results demonstrate that underlying sequence alone can discriminate, to a substantial degree in most cases, regions enriched or depleted for most methylated histone marks.

### Sequence predicts the location of methylated histones genome-wide

**Figure 3:**
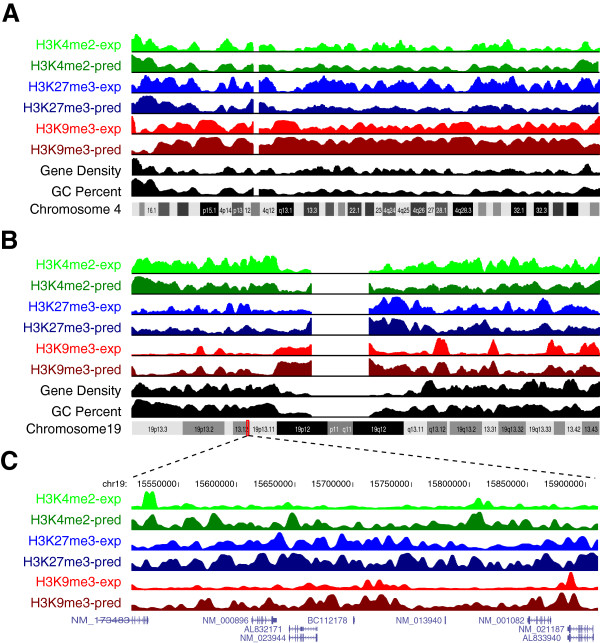
**A comparison between the actual and predicted chromosome-wide locations of H3K4me2, H3K27me3 and H3K9me3.** UCSC genome browser tracks of chromosome-wide predictions (pred, shown as probability) of H3K4me2, H3K27me3 and H3K9me3, compared with experimental data ChIP-SEQ data (exp, shown as tag count [29]), both in 25 kb sliding windows. Both a gene-poor chromosome, chromosome 4 (**A**), and a gene-rich one, chromosome 19 (**B**), are shown. Number of RefSeq genes in every sliding window is shown as a separate “Gene Density” track. In (**C**), details of each histone mark and their predictions are shown for a 400 kb genomic region on chromosome 19, along with all RefSeq genes within this region.

SVM models trained from the above 2,000 selected sequences for each histone mark also perform consistently on the remaining regions in the genome (Additional file 3: Table S3), indicating that they generalize well on all known TSS regions and the non-repetitive portion of the genome. We next trained a single model for each histone mark (with accuracy >75%), using both TSS and non-genic regions, and applied these models to the entire genome, including all repetitive regions, to locate enriched regions for these histone marks. Notwithstanding the fact that ~20% of the regions are not unique [31] and thus were absent from the Barski *et al.* ChIP-Seq experiments, we observe a striking resemblance between experimentally-determined histone profiles and the predicted ones based solely on sequence, again exemplified by H3K4me2, H3K27me3 and H3K9me3 on both a gene-poor chromosome (chromosome 4, Figure 3A) and a gene-rich chromosome (chromosome 19, Figure 3B). The predictions for these three marks across all chromosomes are shown in Additional file 4: Figure S1. Zooming in on a 400-kb window on chromosome 19 reveals that the predicted locations of these marks agree very well with experimental data on a local scale (Figure 3C). In many cases, the prediction probability even reflects the level of enrichment despite the fact that level information was not used in determining the initial binary SVM models (Figure 3 and Additional file 4: Figure S2). Notably, H3K4me2- and H3K27me3-enriched region density positively correlates with both gene density and GC content, while H3K9me3 was just the opposite (Figure 3). These results further extend the association between sequence and methylated histone marks to a genome-wide scale.

### Genomic regions occupied by different methylated histones define clusters that distinguish genic and non-genic regions of the genome

It has been shown that different modifications of histones can promote or interfere with one another [32,33], resulting in co-occupancy of some marks in the same genomic region, while others are mutually exclusive. Alternatively, histone-modifying enzymes may recognize similar or different sequence signals among chromosomal domains. Sequence-based models provide a powerful tool to explore these possible inter-relationships even without complete experimental coverage. Based on the similarity among their occupied sequences, or in other words, the ability to use models trained with one histone type to predict others, we performed cluster analysis on the histone marks for which we had obtained accurate SVM models (cross-validation rate > =75%). By cluster analysis, the 16 methylated histones in TSS regions fall into three groups (Additional file 1: Figure S3A). Many mono-methylated histones, as well as H3K4me2-3 and H3K79me3, are associated with a similar set of TSS regions (Additional file 4: Figure S3A and Additional file 5: Table S4), consistent with the fact that many such histone modifications are associated with active transcription [15]. Notably, these sequences are very different from the two distinct sequence clusters occupied by repressive marks, H3K9me2-3 and H3K27me2-3. The two repressive marks can also be readily distinguished from each other based on local sequence, which again is consistent with existing experimental data on a broad scale [34]. Interestingly, for non-genic regions, H3K9me3 still occupies different sequences from those associated with other histone marks, but H3K27me3-occupied non-genic sequences cannot be distinguished from those occupied by marks of active chromatin (Additional file 4: Figure S3B). These results suggest that whatever mechanisms are responsible for placing these marks in the genome are either directly or indirectly dependent on genomic sequence. Further, the data indicate that some of these mechanisms must be different for genic and non-genic regions.

### Sequence features associated with predictions of methylated histone patterns

While the above analyses all support the existence of predictive genomic characteristics underlying methylated histone patterns in the human genome, they do not by themselves identify the particular sequences involved. We therefore next explored the specific sequence features supporting each of the above SVM classifications to address the nature of the sequence bias.

**Figure 4:**
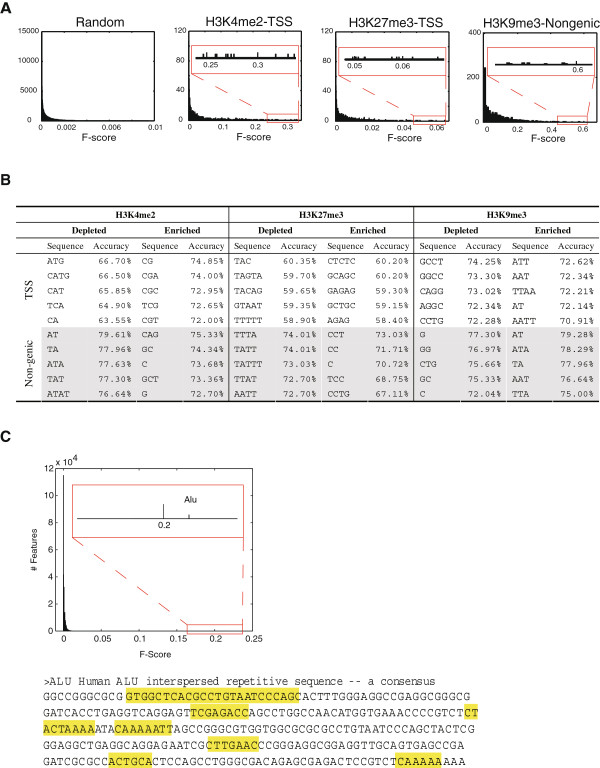
**Highly predictive sequence features.** (**A)** The distribution of F-scores of 1,364 sequence features for three representative histone marks (top 10 features are shown in boxes). A null distribution of F-score (Random) is based on same sequence features from 100 random sets of 1,000 TSS regions. (**B)** Highly predictive sequence features for H3K4me2, H3K27me3 and H3K9me3 and their SVM classification accuracy as single features. (**C)** H3K27me3-enriched regions are depleted for Alu retrotransposons. Alu has a much higher F-score than any other repetitive element and all short sequences. The consensus sequence of Alu with highly predictive sequences features highlighted.

For each sequence feature, we calculated an F-score, a parameter that considers both within- and between-class variations to estimate their individual discriminating power [35]. A sequence feature with a high F-score has large between-class variation and/or small within-class variation and therefore is more likely to be discriminative. (Used in this way, sequence feature F-scores are conceptually similar to F_st_ values used in population genetics to describe allelic variants that can distinguish different population groups.) In general, the F-scores calculated from random sequence datasets are extremely small (<< 0.01, Figure 4A). For any given methylated histone dataset, most features have small F-scores and therefore likely contribute little, if at all, to classification. Relatively few sequences have high F-scores (Figure 4A); these likely correspond to the sequence features responsible for accurately predicting the observed histone patterns.

To overcome the dataset dependency nature of F-score calculations, we selected sequence features that consistently have the highest F-scores across multiple, independent subsets of sequences. The Pearson linear correlation coefficients between each feature and whether or not a region is enriched for a particular histone mark were also computed to reveal their degrees of association. Finally, these consistent features were assessed by SVM to evaluate their discrimination power as single features (Supplemental Table S5). On the basis of this analysis, highly discriminative sequences in TSS regions display three notable features: regions marked by methylated histones that are associated with active transcription (e.g. H3K4me2) are enriched for CpG-containing sequence features but are depleted for AT-rich sequences; H3K27me3 regions are enriched in poly-purines and poly-pyrimidines, but are depleted in AT-rich features; H3K9me2 and H3K9me3 regions are enriched for AT-rich features but depleted in GC-rich ones (Figure 4B and Additional file 6: Table S5). In contrast, the patterns are much simpler among non-genic regions: GC-richness is associated with all the methylated histones except regions enriched in H3K9me3, in which the association is the opposite (Figure 4B). These features collectively reveal, at least to a first-order, the trends of the genomic bias for methylated histone patterns, based on which epigenetic information can be accurately inferred from primary sequence information.

For H3K27me3, the majority of short sequences that have high F-scores are parts of human Alu repetitive elements, SINE non-LTR retrotransposons that comprise at least 10.8% of the human genome (calculated from UCSC genome browser). To systematically investigate the potential contribution to the genomic code of all known repetitive elements, each of which can be viewed as a unique combination of shorter sequences, we performed F-score and SVM analysis among a training set including 2,000 TSS regions. The Alu family is the only repetitive element whose content has an unusually high F-score and by itself it predicts ~70% of training samples (Figure 4C). In addition, Alu content is negatively correlated with H3K27me3 enrichment (corr = −0.42). This is consistent with a previous report that H3K27me3-enriched regions in mouse ES cells are relatively depleted for transposons [24]. This observation is clearly not just a reflection of the absence of repeat-associated sequences from ChIP experiments, because other repetitive sequences do not have discriminative power, nor does Alu distinguish H3K4me2 and H3K9me3 in TSS regions (not shown). As H3K27me3 represents a repressive mechanism for controlling the expression of genes [24,36,37], it is likely that Alu insertions into vicinities of these genes are selectively eliminated during evolution to ensure gene function in development. Consistently, Alu elements have been found to be excluded from tissue-specific genes but enriched in housekeeping genes, a genomic trend apparent both at the level of individual genes and at the level of megabase-sized chromosome bands [38-41].

It is possible that SVM models simply classifies GC/AT content, as there is a significant GC bias in the features listed in Table S5. To test this hypothesis, we first randomly permuted the base order of selected regions to preserve the base composition but to randomize all higher *k*-mer (*k* = 2,3,4,5) content (“singlet permutation”; see Methods for a description of the parameters altered in these permutation experiments). For H3K9me3 in TSS regions, base composition seems to be the only key factor for accurate prediction, because none of the permutations tested significantly affected the prediction accuracy (Additional file 4: Figure S4). In contrast, prediction on these randomized sequences for H3K4me2 and H3K27me3 in TSS regions was completely lost, with the models classifying all regions to be enriched (H3K4me2) or depleted (H3K27me3) regardless of the original labels (Additional file 4: Figure S4).

To explore further the apparent dependence of H3K4me2 and H3K27me3 predictions on sequence content, we permuted the TSS sequences to preserve both base composition and dinucleotide frequencies, but altering all higher *k*-mer content (“doublet permutation” in Additional file 4: Figure S4). We observed a significant restoration in prediction rate for H3K4me2, likely due to the restoration of correct CpG content, since prediction was lost in a control experiment when we only permuted the content of CpG-containing sequence features while keep everything else the same (Additional file 4: Figure S4). In contrast, while addition of the correct dinucleotide content partially restored H3K27me3 prediction, this appears to be independent of CpG content, since CpG permutation itself has little effect. These findings suggest that models for H3K27me3 recognize CpG-independent, higher-order (*k* > 2) sequence features while models for H3K4me2 recognize CpG-dependent features and models for H3K9me3 depend only on base composition, not appreciably on content. Combined, these results are consistent with the highly predictive features identified based on F-scores and suggest that the observed sequence bias for histone modification exists at multiple levels.

### A sequence bias for histone modification is likely a common theme for the human genome

**Figure 5:**
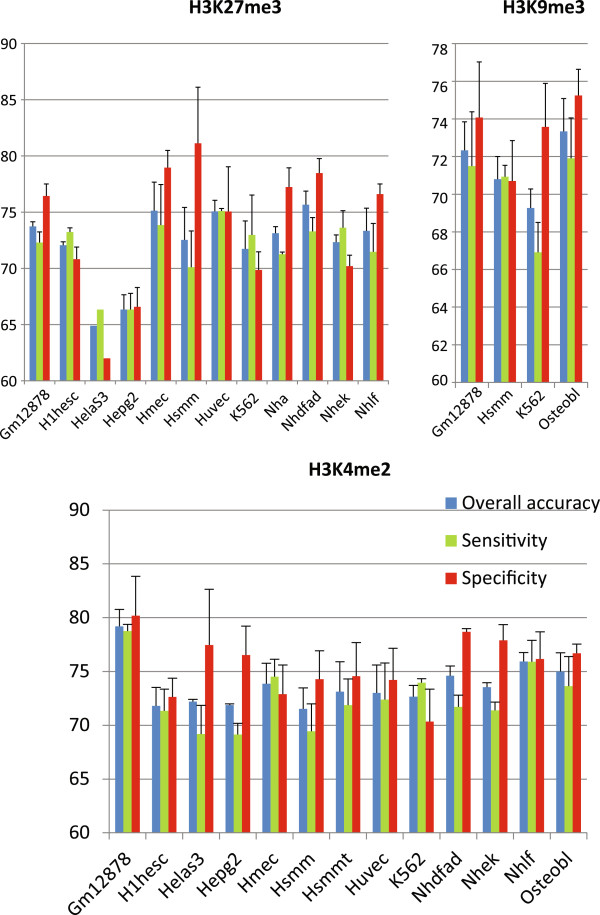
**SVM classification of epigenetic marks in ENCODE human cell lines.** For all three histone modification data sets the overall classification accuracy (blue), sensitivity (true positive rate, green) and specificity (true negative rate, red) from three independent replicates were shown. The names of the cell lines are on the x-axis.

To test whether genomic sequence also influence histone modifications in other tissues and samples, similar SVM experiments were carried out with ChIP-Seq data from the ENCODE project [42]. Briefly, enriched and depleted TSS regions were selected for three histone marks across a number of cell lines used by ENCODE investigators (11 lines for H3K27me3, 13 lines for H3K4me2 and three lines for H3K9me3), followed by SVM training/predicting the same way as described above. Consistent with the observation made with data from CD4+ cells, SVM models based on genomic sequence features are generally able to predict the histone modification status in the same cell line, with an accuracy ranging from 65% to 75% across 27 different data sets (Figure 5 and Additional file 7: Table S6). SVM models trained with one cell line can also largely predict the histone marks from other cell lines, although often at slightly lower rates (Additional file 7: Table S6).

## Discussion

Our results establish a strong association between the primary DNA sequence and an extensive set of histone methylation marks in multiple human cell lines, supporting the hypothesis that at least part of the underlying basis for the histone code is encoded in the genome. This study also provides an example of how genome organization and sequence might directly impact biological function(s). Furthermore, the ability of sequence models to make such predictions provides insights into the establishment and maintenance of epigenetic modifications in complex genomes.

It is worth noting that, for several reasons, the prediction accuracy of sequence-based models for the histone code as outlined here is likely an underestimate. First of all, we have considered only the linear combination of short *k*-mers in local regions. Future analyses using additional information in the sequence (e.g. non-linear combinations of *k*-mers, spatial relationships of *k*-mers within the genome, etc.) will be required to further explore the predictive potential of the genomic sequence and to thus further define the genomic code [43]. Second, SVM models tend to give higher probability to genomic regions that have high levels of a particular epigenetic mark, even without such information being provided in the training sets. Supplying quantitative information to the training sets will likely enable the models to make more accurate predictions. Finally, some “false” predictions may arise from the noise of the experimental approaches, and inclusion of additional datasets may help identify and thus reduce that noise.

A nucleosome code has been described for nucleosome organization and positioning in yeast and *C. elegans*[44-46]. Such a nucleosome code could influence or contribute to our selection of regions in the human genome enriched/depleted for many histone marks in the same way as in yeast [47]. However, nucleosome density appears to be largely invariable except for limited changes in response to signaling or development [14,48]. Therefore, although nucleosome density data that might serve as a control for our analysis are unavailable, our conclusions appear unlikely to be biased by nucleosome placement. Furthermore, most of our analyses are based on several kilobases of sequence containing many nucleosomes, which should effectively reduce the bias even if there is a local nucleosome positioning code in complex genomes. In addition, by separately analyzing TSS and non-genic regions we avoided potential bias from features specifically associated with transcription.

How might primary genomic sequence influence epigenetic modification(s)? Certain types or combinations of sequences could form higher-order signals with specific conformation, which might be used to recruit histone-modifying enzymes and/or to facilitate their spreading in *cis* to form the initial epigenetic framework. Different sequence composition may target, or have different affinity for, different chromatin remodeling complexes [49,50]. Other sequence-specific events, including transcription and chromatin remodeling factors, could further tailor this framework to create specific and dynamic epigenetic patterns downstream during development, in ways not necessarily dependent on sequence in that later context. The dataset evaluated here thus provides only a single “snapshot” of epigenetic modification in time, reflecting the combined results of genomic sequence influence and subsequent cell type-specific chromatin remodeling. Notwithstanding the proposed general nature of the genomic bias reported here, the situation is no doubt further complicated at specific loci or in specific regions by various genomic features such as insulators and barriers, replication origins, or centromeres and telomeres.

A prerequisite for the genomic influence hypothesis is that the enzymes responsible for establishing epigenetic marks recognize subtle structural difference of underlying genomic sequence. Consistent with this concept, it has been recently shown that Dnmt3a, an enzyme that catalyzes *de novo* CpG methylation, recognizes CpG periodicity signals encoded in the genome [51]. Histone-modifying enzymes could similarly utilize structural information, but not necessarily the same information, because our preliminary results indicate that CpG periodicity is not important for prediction methylated histone marks (data not shown). GC- and AT-rich sequences have been shown to differ in chromatin conformation as well as histone modifications in yeast [52], which further strengthens the link between sequence and histone modifications. Further explorations of the information encoded in the genome, especially sequence features discovered in this study, and a deeper understanding of the structural hierarchies encoded in the genome, should help to better inform how the genome sequence is interpreted or “read” by epigenetic factors.

Conceptually, sequence could be the major driving force influencing the epigenetic framework, which, if true, could help explain the persistence of genome-scale epigenetic modifications and the observed clustering of tissue-specific or generalized functions in complex genomes. For maintaining “housekeeping” functions across different cell types, for example, it would seem to be much less expensive to use the genomic sequence instead of complicated and error-prone gene regulatory networks. This hypothesis predicts that the epigenetic framework should be largely invariant in cells from different developmental contexts. Indeed, this prediction is supported by recent genome-scale profiling studies of several histone marks in embryonic stem cells and lineage-committed cells [53-55]. The specific and dynamic epigenetic patterns that have been demonstrated during development and the cell cycle [19] appear to be at a much smaller and more local scale than the overall framework laid down by sequence features across the genome.

## Conclusion

This study demonstrates a strong association between the primary DNA sequence of the human genome and an extensive set of histone methylation marks described in multiple human cell lines and thus supports the hypothesis that at least part of the underlying basis for the histone code is encoded in the genome. These findings illustrate how genome organization and sequence might establish and maintain epigenetic modifications in complex genomes and thereby directly impact biological function(s).

## Methods

### Enriched and depleted regions for histone marks in human CD4+ T-cells

#### Statistics for null distribution

In order to select enriched regions, we first simulated a null distribution based on the assumption that the number of sequence tags observed in a region with defined size follow a Poisson distribution [56]. Based on this null distribution, two cutoff counts were made at 1% and 99%, thus only allowing 2% False Discovery Rates (FDR).

#### Regions surrounding known Transcription Start Sites (TSS)

We considered ±2 kb sequence flanking the TSS of all known RefSeq genes (UCSC hg18). Duplications were subsequently eliminated to yield 19,812 unique regions. For each histone modification, enriched regions were defined to have tag counts greater than the 99% line (based on the null distribution, above), while depleted regions have tag counts less or equal to the 1% cutoffs. The other regions were considered “neutral” and were not analyzed further (see Figure 2A).

#### Non-genic regions

To identify a set of non-genic regions for comparison with TSS regions defined above, we first selected genomic regions that are (1) not within 100 kb of any RefSeq genes; (2) free of known repetitive sequences and (3) >1.5 kb. This resulted in 43,039 regions from the current UCSC human genome assembly (hg18). These regions were further divided into enriched, depleted, or neutral regions in the same way as the TSS regions, except that the 1% and 99% cutoff counts were normalized to the size of each region.

### Sequence feature extraction

Unless specified, DNA sequences were extracted from UCSC assembly (hg18), and *k*-mer (*k* = 1,2,3,4,5) content (count/size) was calculated for each region, yielding a total of 1,364 sequence features to represent the underlying sequence information of a particular genomic region. For models with repeat features, the content of repeat families as well as individual repeats were extracted from UCSC annotations.

### Support Vector Machine (SVM) training and testing

LibSVM version 2.84 software (http://www.csie.ntu.edu.tw/~cjlin/libsvm/) was used for SVM classification. With the assumption that the data used in this paper are linearly separable, we used linear SVM models throughout the analysis. In a typical classification experiment, a proportion of the regions were selected randomly from each dataset for training and cross validation purposes, while an independent set was used for testing. Such training/testing sessions were performed 10 times and *p*-values were based on paired *t*-tests.

### Feature selection

Briefly, we selected 100 features with highest F-scores [35] for each 100 randomly sampled regions that were either enriched or depleted for a specific epigenetic mark. The above sampling/feature selection process was repeated 100 times and features that were selected more than 30 times were defined as highly discriminative features. These features were tested for their correlation with whether or not the region is enriched and for their SVM classification performance as single features.

### Whole-genome prediction for epigenetic marks

For whole-genome prediction, we used sliding windows of 2.5 kb in size with 2 kb overlaps to cover the entire human genome. To predict the probability for each window to be bound by an epigenetic mark of interest, we used SVM models based on 2,000 regions enriched for a histone mark (1,000 non-genic regions and 1,000 TSS regions, for both enriched and depleted) with 1,364 sequence features. The resulting predictions were made into local UCSC genome browser tracks and visualized along with other genomic information.

### Cluster analysis of regions occupied by different modified histones

Using datasets from Barski *et al.*[29], SVM models were trained with 2,000 regions for each histone mark, and those with a 10-fold cross-validation rates greater than 75% were used to predict other histone marks, excluding H3K79me2 (too few regions). The dissimilarity in their occupied sequence between any two histone marks, or distance, was defined as the average misclassification rates of their mutual predications. Such distances were subsequently used to perform hierarchical cluster analysis. Dendrograms, as in Additional file 4: Figure S2, were drawn with default color threshold (0.7) for coloring clusters. Clustering analysis was performed using MATLAB™ software version 7.3.0.

### Singlet, doublet sequence and CpG permutations

Singlet- and doublet-sequence permutations were performed by shuffling the positions of either single nucleotides or dinucleotides while maintaining constant the base composition or the dinucleotide frequency of the original sequences, respectively. Feature extraction was then performed on the permuted sequences. Since it is not possible to permute CpG only without affecting other sequence motifs, we permuted the frequency of all CpG-containing motifs among the sequences to simulate the effects of CpG permutation. A necessary side-effect of CpG permutation is that the sum of all *k*-mer frequencies within individual sequences may not be precisely 1. However, since these values are usually very small and the direction of change (increase or decrease) for each *k*-mer will be random, the sum is likely to be very close to 1 after the permutation.

## Competing interests

Both authors declare that they have no competing interests.

## Supplementary Material

Additional file 1 **Table S1.** Summary of selected regions enriched/depletedfor histone marks from human CD4+T-cells. Click here for file

Additional file 2 **Table S2.** Number of samples used for SVM training/testing in human T-cells. Click here for file

Additional file 3 **Table S3.** SVM classification for histone marks in human T-cells. Click here for file

Additional file 4 **Figure S1,S2,S3 and S4.** Figure S1. Genome-wide predicted locations of H3K4me2, H3K27me3, and H3K9me3 correlate with experimentally determined profiles in the human CD4 T-cells. Figure S2. Genome-wide predicted locations of H3K4me2, H3K27me3, and H3K9me3 correlate with experimentally determined pro_les in the human CD4 T-cells. Only data from chr10 is shown as an example since plots obtained from the rest of the chromosomes look almost identical as chr10. Each data point corresponds to the experimentally deterimined modi_ed histone enrichment level (x-axis) in a 2.5kb region and the prediction probability by SVM models (y-axis). Enrichment level 6 stands for >2^6 (64 reads per kb), 5 stands for 2^5-2^6, or (52-64), and so on. Red bars in each boxplot indicate median values, and red pluses indicate outliers. As enrichment levels go down, the number of regions predicated to be enriched also go down. Figure S3. Cluster analysis of regions occupied by different epigenetic marks. The hierarchical cluster of histone marks in (a) TSS regions and (b) non-genic regions, based on dissimilarities in their occupied genomic- sequence (measured by SVM misclassification rates). Figure S4. Sequence permutations and their e_ects on classi_cation. Prediction accuracy of SVM models (trained with original sequences, circles) for singlet (triangles), doublet (diamonds) or CpG (squares) permuted sequences. Sensitivity represents the ability to predict enriched regions, and speci_city for depleted regions of a particular methylated histone mark. Click here for file

Additional file 5 **Table S4.** Predictions between epignetic marks using SVM models with high cross-validation accuracy(>75%). Click here for file

Additional file 6 **Table S5.** Features with consistently high F-scores in multiple rounds of classifications, TSS regions. Click here for file

Additional file 7 **Table S6.** SVM classification on ENCODE cell lines for H3K9me3, H3K27me3, H3K4me2. Click here for file
