# Supplementary material for: Evidence for sequence biases associated with patterns of histone methylation
Source: BMC Genomics. 2012 Aug 2;13:367. doi: 10.1186/1471-2164-13-367 (PMC3532361; doi:10.1186/1471-2164-13-367)
Supplement: Additional file 4 — Figure S1,S2,S3 and S4. Figure S1. Genome-wide predicted locations of H3K4me2, H3K27me3, and H3K9me3 correlate with experimentally determined profiles in the human CD4 T-cells. Figure S2. Genome-wide predicted locations of H3K4me2, H3K27me3, and H3K9me3 correlate with experimentally determined pro_les in the human CD4 T-cells. Only data from chr10 is shown as an example since plots obtained from the rest of the chromosomes look almost identical as chr10. Each data point corresponds to the experimentally deterimined modi_ed histone enrichment level (x-axis) in a 2.5kb region and the prediction probability by SVM models (y-axis). Enrichment level 6 stands for >2^6 (64 reads per kb), 5 stands for 2^5-2^6, or (52-64), and so on. Red bars in each boxplot indicate median values, and red pluses indicate outliers. As enrichment levels go down, the number of regions predicated to be enriched also go down. Figure S3. Cluster analysis of regions occupied by different epigenetic marks. The hierarchical cluster of histone marks in (a) TSS regions and (b) non-genic regions, based on dissimilarities in their occupied genomic- sequence (measured by SVM misclassification rates). Figure S4. Sequence permutations and their e_ects on classi_cation. Prediction accuracy of SVM models (trained with original sequences, circles) for singlet (triangles), doublet (diamonds) or CpG (squares) permuted sequences. Sensitivity represents the ability to predict enriched regions, and speci_city for depleted regions of a particular methylated histone mark. [file 1471-2164-13-367-S4.pdf]

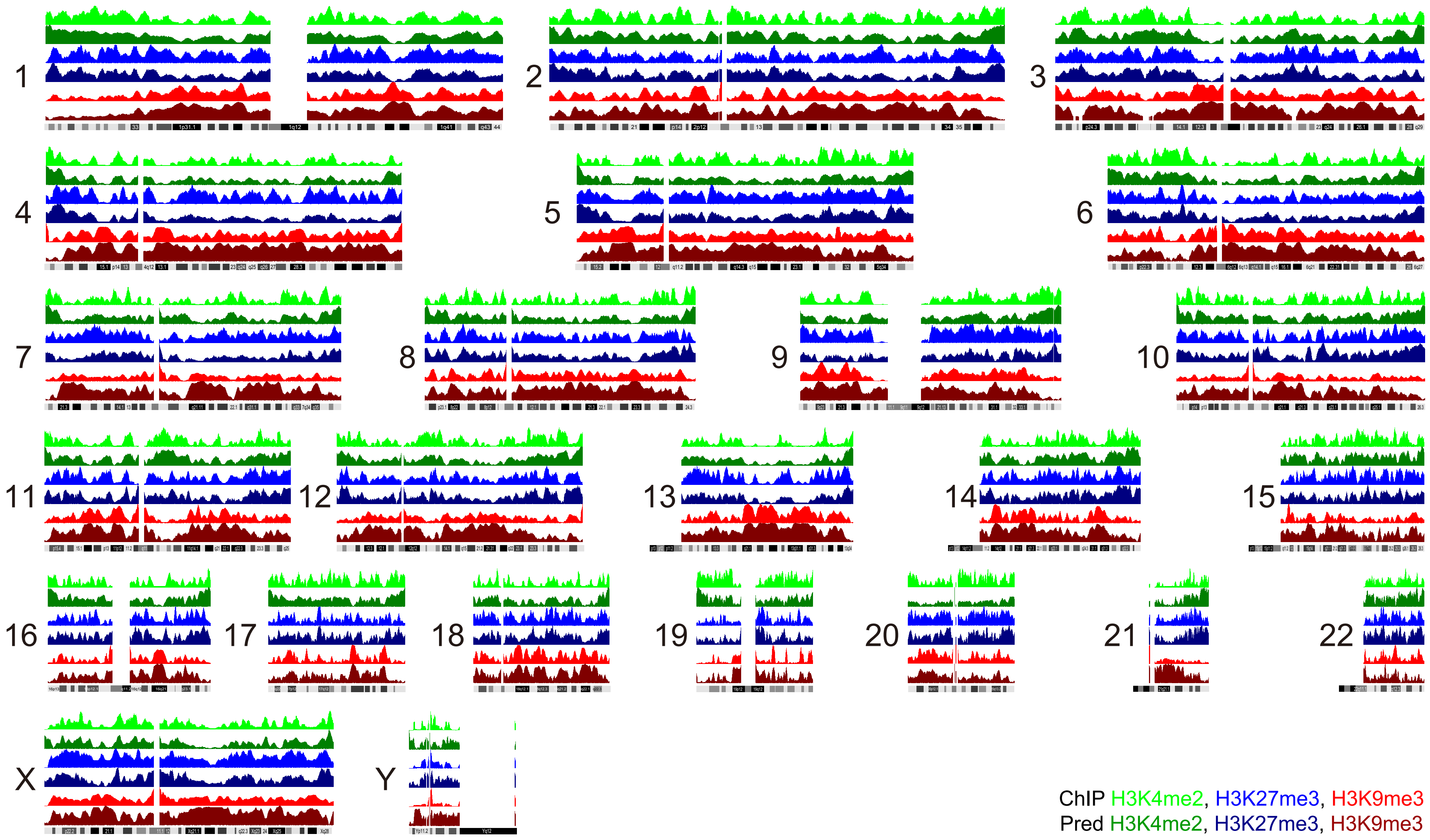

Figure S1. Genome-wide predicted locations of H3K4me2, H3K27me3, and H3K9me3 correlate with experimentally determined profiles in the human CD4 T-cells.

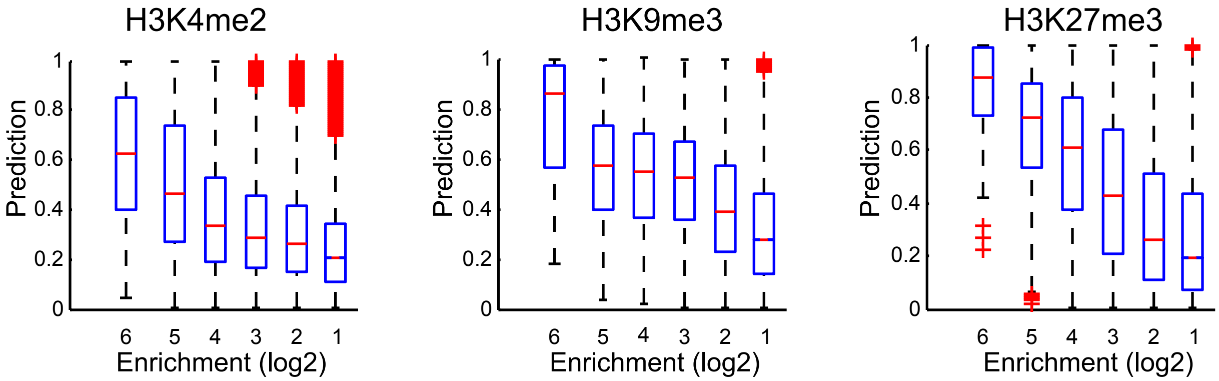

Figure S2. Genome-wide predicted locations of H3K4me2, H3K27me3, and H3K9me3 correlate with experimentally determined profiles in the human CD4 T-cells. Only data from chr10 is shown as an example since plots obtained from the rest of the chromosomes look almost identical as chr10. Each data point corresponds to the experimentally deterimined modified histone enrichment level (x-axis) in a 2.5kb region and the prediction probability by SVM models (y-axis). Enrichment level 6 stands for >2^6 (64 reads per kb), 5 stands for 2^5-2^6, or (52-64), and so on. Red bars in each boxplot indicate median values, and red pluses indicate outliers. As enrichment levels go down, the number of regions predicated to be enriched also go down.

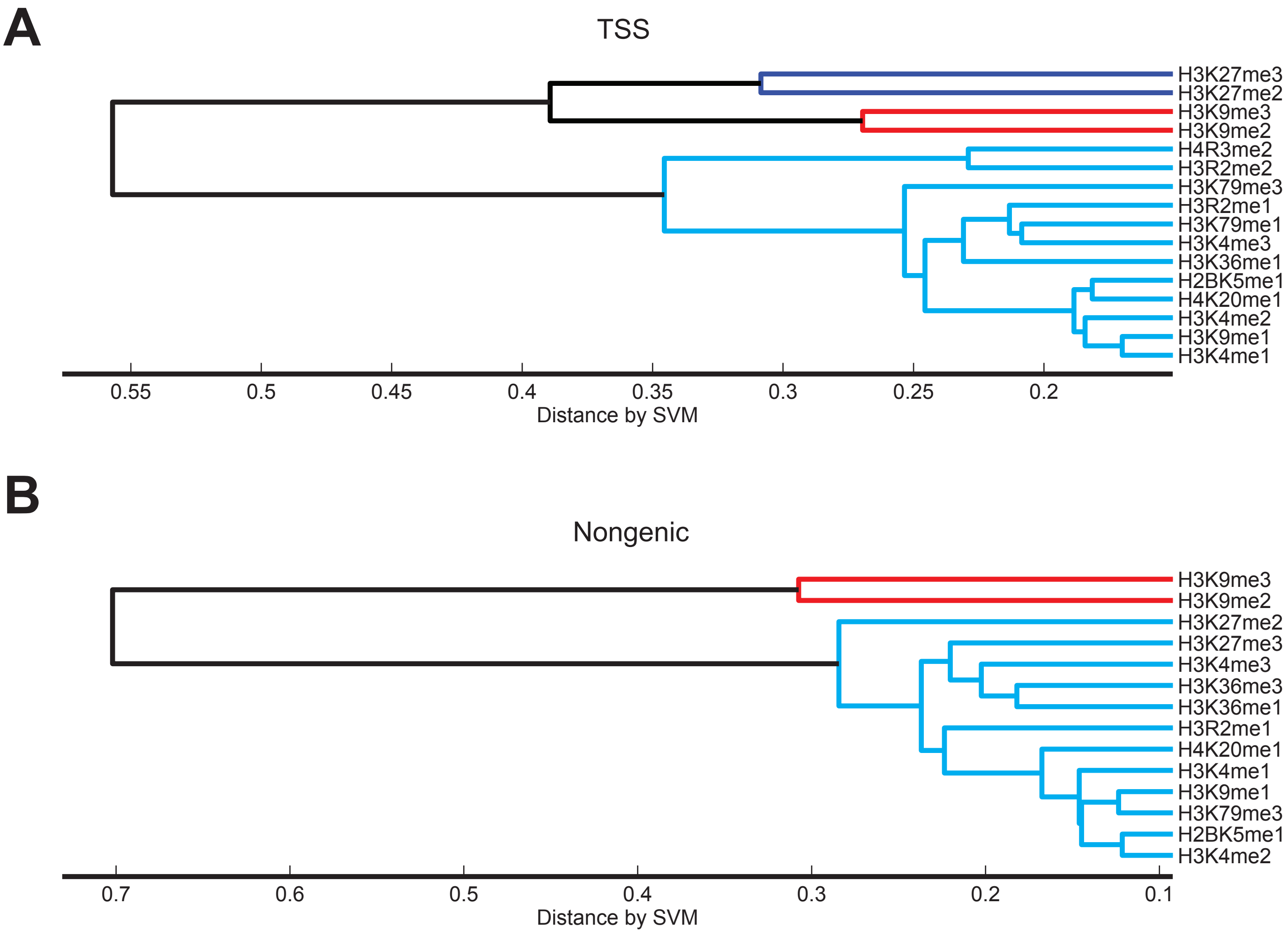

Figure S3. Cluster analysis of regions occupied by different epigenetic marks.
The hierarchical cluster of histone marks in (a) TSS regions and (b) non-genic regions, based on dissimilarities in their occupied genomic sequence (measured by SVM misclassification rates).

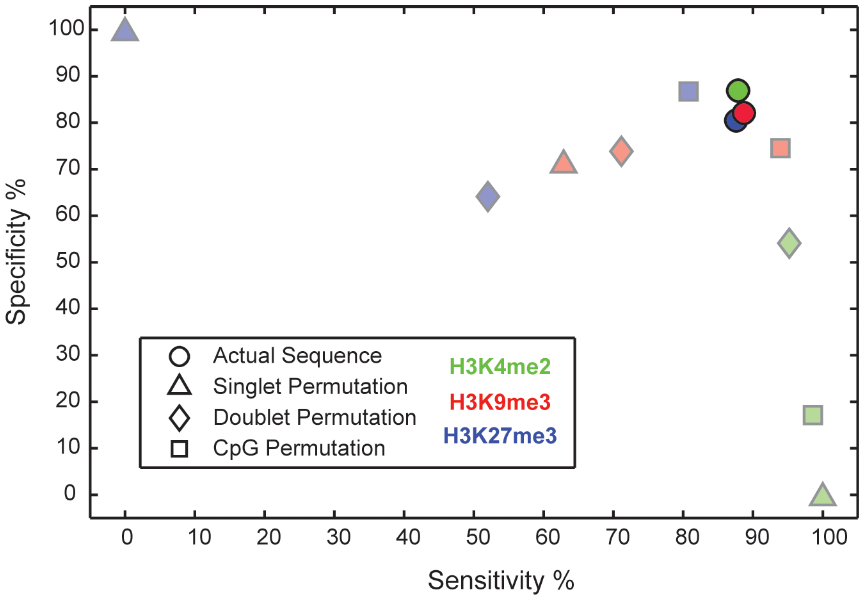

Figure S4. Sequence permutations and their effects on classification. Prediction accuracy of SVM models (trained with original sequences, circles) for singlet (triangles), doublet (diamonds) or CpG (squares) permuted sequences. Sensitivity represents the ability to predict enriched regions, and specificity for depleted regions of a particular methylated histone mark.
